# Isolated ST-Elevation Myocardial Infarction Involving Leads I and aVL: Angiographic and Electrocardiographic Correlations from a Tertiary Care Center

**DOI:** 10.1155/2021/7638020

**Published:** 2021-06-21

**Authors:** Abhishek Singh, Sudhanshu Dwivedi, Akshyaya Pradhan, Varun S Narain, Rishi Sethi, Sharad Chandra, Pravesh Vishwakarma, Gaurav Chaudhary, Monika Bhandari, Akhil Sharma

**Affiliations:** ^1^Department of Medicine, King George's Medical University, Lucknow 226003, Uttar Pradesh, India; ^2^Department of Cardiology, King George's Medical University, Lucknow 226003, Uttar Pradesh, India

## Abstract

**Background:**

Determining the infarct-related artery in STEMI during a coronary angiogram can be challenging due to the affliction of multiple vessels. Isolated STEMI involving only EKG leads I and aVL is infrequent. Localization of infarct-related artery based on EKG findings has not been previously done in this subset.

**Methods:**

All consecutive de novo acute coronary syndrome (ACS) patients admitted to coronary care unit with ST elevations involving only leads I and aVL were screened for enrollment. Patients with ST elevation in any additional lead and those who refused a coronary angiogram were excluded. Subsequently, a coronary angiogram was done as part of primary PCI or a pharmacoinvasive approach to identify the infract-related artery (IRA). IRA was defined by characteristics of lesion, flow of blood through stenosis, and presence of intracoronary thrombus. Coronary angiogram was interpreted by two independent observers blinded to the EKG findings. ST changes in inferior and precordial leads were analyzed to find ECG predictors of the culprit artery.

**Results:**

A total of 54 eligible patients of ACS were included in the study. The first major diagonal (D1) was the most frequent IRA in 35.2% followed by left circumflex-obtuse marginal (LCX-OM11) in 29.6%, left anterior descending (LAD) in 20.4%, and ramus intermedius (RI) in 14.8%. Out of total patients with ST depression in lead V2, the LCX-OM11 group was IRA in 50% cases while the RI, D1, and LAD groups accounted for 31.8%, 13.6%, and 4.5%, respectively (*p* < 0.001). Similarly, LCX-OM1 was the most frequent IRA subjects with ST depressions in leads V1 and V3 (44.4%; *p* = 0.010 and 46.2%; *p* = 0.003, resp.). On the contrary, in patients with ST depression in lead III, LAD and D1 were the most frequent IRA as compared to LCX-OM1 and RI though statistical significance was not attained (*p* = 0.857 for lead III). ST-segment depression in lead V2 had a positive predictive value of 60% and a negative predictive value of 100% for LCX-OM1 as IRA. Similarly, ST-segment depression in lead V2 had a positive predictive value of 20% and a negative predictive value of 100% for the RI group.

**Conclusions:**

In patients presenting with isolated ST elevation in leads I and aVL, the most frequent IRA on angiogram was first diagonal. ST depressions in EKG leads V1–V3 were the most common predictor of LCX–OM1 while those in inferior leads indicated LAD-D1 as the IRA.

## 1. Introduction

Multivessel disease is a common finding in patients undergoing primary PCI for ST-Elevation Myocardial Infarction (STEMI) [1]. Identifying the infarct-related coronary artery during coronary angiography is of paramount importance as guidelines emphasize on the treatment of the culprit artery first followed by management of noninfarct vessels later unless complicated by cardiogenic shock [2, 3]. This issue may further be compounded by numerous variations in coronary artery anatomy [4]. In order to delineate the infarct-related artery (IRA) in these difficult cases, one may resort to echocardiography, cine-ventriculography, and electrocardiographic changes [5–7].

Two earlier studies have elaborated the EKG changes of ACS due to occlusion of the first diagonal branch [8, 9]. Both these studies showed that ST-segment elevation in lead aVL was the most important EKG finding. A study by Birnbaum et al. described electrocardiographic differentiation between occlusion of LAD, first diagonal, and first obtuse marginal coronary artery in patients having ACS with ST-segment elevation in lead aVL along with any other lead [10]. However, to our knowledge, no study has ever been done which describes the angiographic correlation of patients with ACS and having isolated ST-segment elevation only in leads I and aVL. Hence, we hypothesized that, by identifying different EKG patterns resulting from occlusion of a particular coronary artery and its branches, we will be in a better position to predict IRA in patients with de novo ACS involving ST-segment elevation in leads I and aVL only. The ST-segment depressions in various limb and precordial leads were examined to identify a particular pattern associated with the occlusion of a coronary artery.

## 2. Methods

### 2.1. Patients

**Figure 1:**
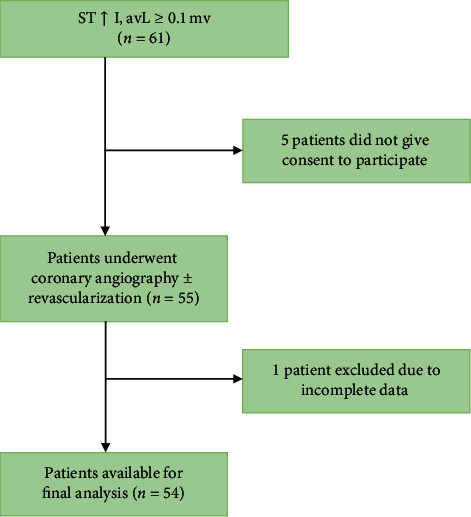
Flow diagram of the study.

Patients admitted to the Department of Cardiology with Acute STEMI from November 2017 to November 2018 were evaluated prospectively for enrollment. Diagnosis of acute myocardial infarction was made on the history of typical chest pain, ST-segment elevation of >0.1 mV only in leads I and aVL and an associated rise and/or fall of hs-cTnT (measured by Cobas e 411, Roche Diagnostics, by the Chemiluminescence method) values with at least one value above the 99^th^ percentile URL [11]. Reperfusion therapy was administered in accordance with the standard Guideline-based criteria. Only patients who underwent coronary angiography within 14 days of AMI were included. Conditions which may cause ST/T changes like pacing, digitalis therapy, left ventricular hypertrophy, and bundle branch block were excluded. The study flow diagram is depicted in Figure 1.

### 2.2. ECG Evaluation

The first standard 12-lead EKG on admission was evaluated by two independent observers blinded to angiographic findings. The EKGs were recorded at a paper speed of 25 mm/sec at a calibration of 1 mV = 10 mm. ST-segment deviation from the isoelectric line, determined by a line drawn between subsequent TP segments, was measured manually to the nearest 0.5 mm in every lead at 0.06 seconds after the J point. The ST segment was considered elevated or depressed if it was −>0.1 mV above or below the isoelectric line, respectively. Otherwise, it was considered isoelectric. For inclusion in the study, the subjects were to have more than 1 mm ST elevation in leads I and aVL but not any other leads. EKG with ST elevations definitely less than 1 mm and upsloping type rather concave upward type typical of a STEMI were excluded.

### 2.3. Coronary Angiography

Selective coronary cineangiography was performed within 14 days of admission. Every coronary angiogram was evaluated by two independent observers blinded to ECG findings. IRA was identified by characteristics of lesions (ulcerated plaque, severe stenosis), flow of blood through stenosis, and presence of intracoronary thrombus.

### 2.4. Statistical Analysis

Categorical variables were presented in number and percentage (%) and continuous variables were presented as mean ± SD. Odds ratio with 95% confidence intervals and sensitivity, specificity, NPV, and PPV were calculated for selected variables and their significance. Qualitative variables were compared using the Chi-Square test/Fisher's exact test as appropriate. A *p* value of <0.05 was considered statistically significant. The data was entered in MS EXCEL spreadsheet and analysis was done using Statistical Package for Social Sciences (SPSS) version 16.0.

## 3. Results

### 3.1. Demographic Profile (Table 1)

The study sample consisted of 54 patients between 23 and 74 years of age, with a mean age of 50 ± 12 years. The majority of the patients were between 51 and 65 years of age. There were 6 patients between 23 and 35 years (11.1%), 21 patients between 36 and 50 years (38.9%), 24 patients between 51 and 65 years (44.4), and the remaining 3 patients above 65 years (5.6%). The sex ratio in our study population showed that the male patient proportion was higher than that of females, that is, 75.9% and 24.1%, respectively. Regarding the therapeutic strategy employed, 66.7% of patients were thrombolyzed with streptokinase and 11.1% of patients underwent primary PCI. The remaining 22.2% of patients were nonthrombolyzed because they were late presenters and did not meet the guideline-based criteria for reperfusion.

### 3.2. Angiographic Profile (Figure 2)

Single-vessel, double-vessel, and triple-vessel diseases were present in 32 (59.3%), 20 (37%), and 2 (3.7%) patients, respectively. The most frequent infarct-related artery (IRA) was first diagonal artery (D1) and left circumflex/1^st^ obtuse marginal (LCX-OM11) artery in 19 (35.2%) and 16 (29.6%) patients, respectively. Left anterior descending (LAD) and ramus intermedius (RI) were IRA in 11 (20.4%) and 8 (14.8%) cases, respectively.

### 3.3. ST Depression as a Predictor of IRA in Overall Study (Table 2)

Out of total patients with ST depression in lead V2, the LCX-OM1 groups accounted for 50% while the LAD, D1, and RI groups accounted for 4.5%, 13.6%, 31.8%, respectively (*p* < 0.001). Among patients with ST depression in lead V5, the D1 group accounted for 31.6% while the LAD, LCX-OM1, and RI groups accounted for 15.8%, 36.8%, and 15.8%, respectively (*p* = 0.904). Meanwhile, in subjects with ST depression in III, the D1 group accounted for 34% while the LAD, LCX-OM1, and RI groups accounted for 20.8%, 30.1%, and 15.1%, respectively.

**Figure 3:**
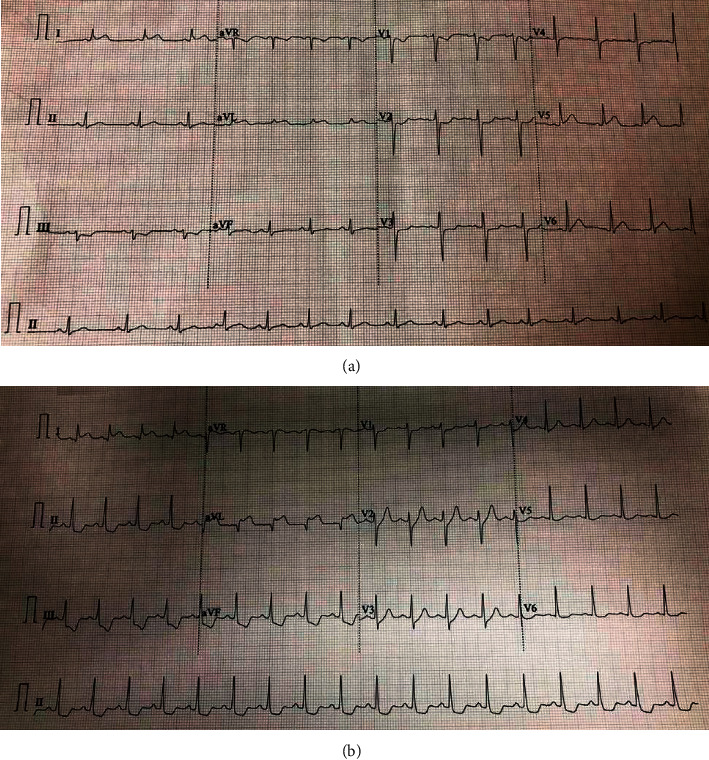
Two examples of admission ECG of patients with AMI with ST elevation in lead I aVL. (a) STEMI from lesion in OM1. ST depression in leads V1 to V3. (b) STEMI from lesion in proximal LAD. ST depression in leads II, III, aVF, V5, and V6. LAD: left anterior descending; OM1: first obtuse marginal.

Hence, ST depression in precordial leads V1–V4 was statistically more common in patients with LCX-OM1 as IRA while ST depression in the inferior lead and lateral leads was more prevalent with LAD/D1 as IRA though not statistically significant.  Figure 3 depicts prototype ECGs features from two different patients with STEMI in culprit lesion in proximal LAD/D1 and OM1, respectively.

### 3.4. ST Depression as a Predictor of IRA in Patients with Single-Vessel Disease (Table 3)

Out of total patients with ST depression in V2, the LCX-OM1 groups accounted for 60% while the LAD, D1, and RI groups accounted for 10%, 10%, and 20%, respectively (*p* < 0.001). Out of total patients with ST depression in V5, the D1 group accounted for 44.4% while the LAD, LCX-OM1, and RI groups accounted for 33.3%, 22.2%, and 15.8%, respectively (*p* = 0.904). In lead III, the LAD (32.3%) and D1 (41.9%) groups accounted for the maximum number of patients as compared to LCX-OM1 (19.4%) and RI (6.5%) groups (*p* = 0.857). ST depression in inferior and lateral leads was more common in the LAD and D1 groups, although it was not statistically significant. Therefore, the pattern of ST depression in single-vessel disease mirrored the finding as seen in the whole study and was not modified by the presence of disease in other vessels.

### 3.5. Use of Lead V2 as Predictor of LCX-OM1 Disease

Using ST depression in lead V2 as a predictor of LCX-OM1 as IRA, the sensitivity and specificity were 100% and 84%, respectively. Moreover, the use of lead V2 as a predictor had an absolute (100%) negative predictive value for LCX-OM1 as culprit vessel while the positive predictive value was only moderate (60%). A separate analysis of ST depression in V2 as a predictor of RI as culprit vessel also mirrored similar findings with 100% negative predictive value of ECG. But owing to the paucity of patients with the RI as culprit, the findings are hypothesis generating only.

## 4. Discussion

STEMI Involving EKG leads I and aVL in isolation is encountered uncommonly in clinical practice. This can arise due to the occlusion of a diagonal branch of LAD, LCX and its branches, and ramus intermedius. Although echocardiography and angiographic features can be of help, EKG criteria remain pivotal for identifying the culprit vessel in this scenario. However, literature is scant with respect to the evaluation of ECG criteria in this context. In a previous attempt, Birnbaum and colleagues concentrated on STEMI with elevation in lead aVL in addition to any other lead. They reported high positive predictive values of ST depression in lead V2 for prediction of OM1 occlusion [10].

However, to the best of our knowledge, the present study is a novel endeavor which describes the angiographic correlation of patients with ACS and having isolated ST-segment elevation only in leads I and aVL. This study demonstrates that ST-segment elevation in leads I and aVL may be caused by occlusion of LAD (proximal to first diagonal), first diagonal branch, first obtuse marginal branch, ramus intermedius branch, and proximal LCX artery.

EKG leads I and aVL face the basal portion of the lateral free wall of the left ventricle. This area is supplied by first diagonal, first obtuse marginal, and ramus intermedius branch. So, AMI with ST-segment elevation in leads I and aVL may represent the involvement of either of these branches [12–16].

The first diagonal was the most frequent culprit artery in the current dataset while Birnbaum et al. had reported that LAD was the most frequent culprit in the majority [10]. Given the inclusion of STEMI ST elevation in other leads in addition to leads I and aVL in their study, the contrasting results are not surprising.

ST-segment depression in inferior leads is commonly found during AMI caused by proximal LAD or diagonal occlusion [17]. These only represent reciprocal changes in lateral/anterolateral infarction. Concurrent with the above-mentioned statement, LAD and 1^st^ diagonal were more frequently the IRA in patients with ST depression in lead III vis-à-vis LCX and RI. Here, our data is consistent with the study done by Birnbaum et al. [10].

Iwasaki et al. described abnormalities in leads I and aVL (but not in the precordial leads) as being most predictive of first diagonal occlusion and particularly in differentiating it from LAD occlusion [8]. The authors used multivariate analysis to compare 34 patients with isolated diagonal occlusion with 20 patients with LAD occlusion (proximal to the first septal perforator branch). Similarly, Engelen et al. in an analysis of 100 acute myocardial infarction (AMI) patients (10 of whom had isolated D1 occlusions) found that ST elevations in leads I and aVL were present in 83% of D1 occlusions; and ST depressions in leads II, III, and aVF were present in 73%, 95%, and 90% of D1 lesions, respectively [18].

All patients in our study had ST elevation in leads I and aVL as inclusion criteria. First diagonal (35.2%) was the most common IRA. LAD proximal to first diagonal was the most common IRA in other studies involving ST elevation in leads I and aVL. LCX-OM1 group accounted for 29.6% of patients. The prevalence of ST-segment depression in lead V2 was 75%, which was in concordance with the previous study done by Birnbaum et al. where the prevalence of ST depression in lead V2 was 64% in the OM group. Our study also showed ramus intermedius as IRA in 14.8% of patients, while none of the patients had ramus intermedius involvement in the study done by Birnbaum et al.

The differential diagnosis among LAD, D1, and OM1 occlusion lies in the EKG changes in the precordial and inferior leads [8, 19]. ST-segment depression in lead V2 had a positive predictive value of 60% and a negative predictive value of 100% for predicting the involvement of LCX-OM1 arteries. ST-segment depression in lead V2 had a poor positive predictive value (20%) and a high negative predictive value (100%) for foretelling involvement of RI as culprit artery. ST-segment depression in lead III had an excellent positive predictive value of 95.83% for the LAD-D1 group as IRA.

Identifying culprit vessel in the setting of high lateral wall MI (diagnosed as ST elevation in leads I and aVL) is of paramount importance as if the culprit vessel is proximal LAD; then, urgent revascularization has to be done [20, 21]. In resource-poor countries, where the peripheral centers do not have facilities for PCI, the prediction of IRA from the admission EKG is a simple and cost-effective tool to select patients for timely referral for PCI. Hence, ST depression in inferior leads (especially lead III) and lead V6 can be utilized as a surrogate marker for LAD involvement and prompt planning for early primary or pharmacoinvasive PCI.

The small sample size could be a potential limitation. However, the previously published studies are also plagued by a small sample size as this is a very select population. Another potential factor could be that many cases of circumflex occlusion may go unnoticed on EKG. The number can be as high as 25%–50% in some studies [13, 22]. This is especially true for patients with acute occlusion of the left circumflex coronary artery. They may present with either: (i) ST-segment elevation in the inferior leads II, III, and aVF and the lateral leads I, aVL, V5, and V6; (ii) ST-segment depression in the precordial leads V1 to V4; (iii) ST-segment elevation in the high lateral leads I and aVL; or (iv) a normal EKG. Data from multiple studies show that at least half of circumflex occlusions do not meet STEMI criteria leading to missing diagnosis and reperfusion.

More recently, an isolated ST elevation in V2 in EKG, in conjunction with ST depression in lead III in patients with lateral STEMIs, has been shown to connote occlusion of a large diagonal branch of LAD. The peculiar arrangement of lead with ST deviations on a 4 × 3 EKG display format has been termed as the “South African Flag” sign [23]. Despite the exclusion of STEMI with ST elevation in leads other than leads I and aVL, the data concurs with that of ours wherein ST depression in lead III (or any other inferior lead) was suggestive of LAD-diagonal occlusion.

Importantly, EKG changes can be dynamic in the initial hours and Lin et al. have shown that De winters and STEMI can evolve into each other [24]. Hence, by using the admission ECG only, we may have excluded some STEMI who could have had presented with De Winter's pattern at admission.

## 5. Conclusions

In patients presenting with AMI involving ST-segment elevation in leads I and aVL, the most frequent culprit vessel was first diagonal. Out of total patients with ST depression in V1–V4, the LCX-OM1 groups accounted for the maximum number of patients. ST depression in lead III was more common in patients with LAD/D1 as IRA. ST-segment depression in lead V2 has a positive predictive value of 60% and a negative predictive value of 100% for the LCX-OM1 group.

## Figures and Tables

**Figure 2:**
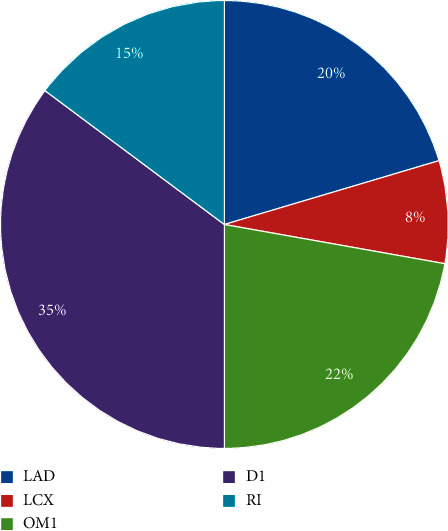
Distribution of infarct-related artery on coronary angiography in the study. LAD: left anterior descending L; LCX: left circumflex; OM1: first obtuse marginal; D1: first diagonal artery; RI: ramus intermedius.

**Table 1:** Demographic, clinical, and angiographic features of the study population.

Demographic and clinical features	Number	Percentage
*Age*		
23 to 35 years	6	11.1
36 to 50 years	21	38.9
51 to 65 years	24	44.4
>65 years	3	5.6

*Gender*		
Male	41	75.9
Female	13	24.1

*Initial therapy*		
Thrombolysis	36	66.7
Primary PCI	6	11.1
Nonthrombolyzed	12	22.2

*Angiographic features*		
Single-vessel disease	32	59.3
Double vessel disease	20	37.0
Triple vessel disease	2	3.7

*Infract-related artery*		
LAD	11	20.4
LCX	4	7.4
OM1	12	22.2
D1	19	35.2
RI	8	14.8

LAD: left anterior descending; LCX: left circumflex; OM1: first obtuse marginal; D1: first diagonal artery; RI: ramus intermedius; PCI: percutaneous coronary intervention.

**Table 2:** Distribution of ST depression in various ECG leads segregated according to the infarct-related artery (IRA) in the overall study (*n* = 54).

ST depression	LAD	LCX	OM1	D1	RI	Total	*p* value
II	9 (23.7)	2 (5.3)	8 (21.1)	11 (28.9)	8 (21.1)	38	0.174
III	11 (20.8)	4 (7.5)	12 (22.6)	18 (34.0)	8 (15.1)	53	0.758
avF	10 (18.9)	4 (7.5)	12 (22.6)	19 (35.8)	8 (15.1)	53	0.408
avR	1 (16.7)	2 (33.3)	0 (0)	1 (16.7)	2 (33.3)	6	0.042*∗*
V1	2 (10.0)	2 (10.0)	7 (35)	3 (15.0)	6 (30.0)	20	0.012*∗*
V2	1 (4.5)	2 (9.1)	9 (40.9)	3 (13.6)	7 (31.8)	22	<0.001*∗*
V3	3 (11.1)	2 (7.4)	9 (33.9)	5 (18.5)	8 (29.6)	27	0.002*∗*
V4	3 (11.5)	2 (7.7)	8 (30.8)	6 (23.1)	7 (26.9)	26	0.031*∗*
V5	3 (15.8)	2 (10.5)	5 (26.3)	6 (31.6)	3 (15.8)	19	0.904
V6	4 (21.1)	1 (5.3)	5 (26.3)	6 (31.6)	3 (15.8)	19	0.970

LAD: left anterior descending; LCX: left circumflex; OM1: first obtuse marginal; D1: first diagonal artery; RI: ramus intermedius; PCI: percutaneous coronary intervention. ^∗^Statistically significant.

**Table 3:** Distribution of ST depression in various ECG leads segregated according to the infarct-related artery (IRA) in patients with single-vessel disease only (*n* = 32).

ST depression	LAD	LCX	OM1	D1	RI	Total	*p* value
II	8 (36.4)	1 (4.5)	4 (18.2)	7 (21.8)	2 (9.1)	22	0.204
III	10 (32.3)	2 (6.5)	4 (12.9)	13 (41.9)	2 (6.5)	31	0.857
avF	9 (29.0)	2 (6.5)	4 (12.9)	14 (45.2)	2 (6.5)	31	0.686
avR	1 (25.00	1 (25.0)	0 (0)	1 (25.0)	1 (25)	4	0.189
V1	2 (22.2)	1 (11.1)	3 (33.3)	1 (11.1)	2 (22.2)	9	0.010*∗*
V2	1 (10)	2 (20.0)	4 (40.0)	1 (10.0)	2 (20.0)	10	<0.001*∗*
V3	3 (23.1)	2 (15.4)	4 (30.8)	2 (15.4)	2 (15.4)	13	0.003*∗*
V4	3 (21.4)	2 (14.3)	4 (28.6)	3 (21.4)	2 (14.3)	14	0.008*∗*
V5	3 (33.3)	1 (11.1)	1 (11.1)	4 (44.4)	0 (0)	9	0.862
V6	4 (44.4)	0 (0)	1 (11.1)	4 (44.4)	0 (0)	9	0.684

LAD: left anterior descending; LCX: left circumflex; OM1: first obtuse marginal; D1: first diagonal artery; RI: ramus intermedius; PCI: percutaneous coronary intervention.

## Data Availability

Data are available from the corresponding author on request.

## References

[1] Park D.-W., Clare R. M., Schulte P. J. (2014). Extent, location, and clinical significance of non-infarct-related coronary artery disease among patients with ST-elevation myocardial infarction. *JAMA*.

[2] Ibanez B., James S., Agewall S. (2017). ESC Guidelines for the management of acute myocardial infarction in patients presenting with ST-segment elevation: the Task Force for the management of acute myocardial infarction in patients presenting with ST-segment elevation of the European Society of Cardiology (ESC). *European Heart Journal*.

[3] O'Gara P. T., Kushner F. G., Ascheim D. D. (2013). ACCF/AHA guideline for the management of ST-elevation myocardial infarction: a report of the American college of Cardiology foundation/American heart association task force on practice guidelines [published correction appears in circulation. *Circulation*.

[4] Levin D. C., Harrington D. P., Bettmann M. A., Garnic J. D., Davidoff A., Lois J. (1982). Anatomic variations of the coronary arteries supplying the anterolateral aspect of the left ventricle. *Investigative Radiology*.

[5] Popma J. J., Braunwald E. (2012). Coronary arteriography. *Braunwald’s Heart Disease: A Textbook of Cardiovascular Medicine*.

[6] Blanke H., Cohen M., Schlueter G. U., Karsch K. R., Rentrop K. P. (1984). Electrocardiographic and coronary arteriographic correlations during acute myocardial infarction. *The American Journal of Cardiology*.

[7] Zimetbaum P. J., Josephson M. E. (2003). Use of the electrocardiogram in acute myocardial infarction. *New England Journal of Medicine*.

[8] Iwasaki K., Kusachi S., Kita T., Taniguchi G. (1994). Prediction of isolated first diagonal branch occlusion by 12-lead electrocardiography: ST segment shift in leads I and aVL. *Journal of the American College of Cardiology*.

[9] Sclarovsky S., Birnbaum Y., Solodky A., Zafrir N., Wurzel M., Rechavia E. (1994). Isolated mid-anterior myocardial infarction: a special electrocardiographic sub-type of acute myocardial infarction consisting of ST-elevation in non-consecutive leads and two different morphologic types of ST-depression. *International Journal of Cardiology*.

[10] Birnbaum Y., Hasdai D., Sclarovsky S., Herz I., Strasberg B., Rechavia E. (1996). Acute myocardial infarction entailing ST-segment elevation in lead aVL: electrocardiographic differentiation among occlusion of the left anterior descending, first diagonal, and first obtuse marginal coronary arteries. *American Heart Journal*.

[11] Thygesen K., Alpert J. S., Jaffe A. S. (2019). Fourth universal definition of myocardial infarction. *European Heart Journal*.

[12] Movahed A., Becker L. C. (1984). Electrocardiographic changes of acute lateral wall myocardial infarction: a reappraisal based on scintigraphic localization of the infarct. *Journal of the American College of Cardiology*.

[13] Huey B. L., Beller G. A., Kaiser D. L., Gibson R. S. (1988). A comprehensive analysis of myocardial infarction due to left circumflex artery occlusion: comparison with infarction due to right coronary artery and left anterior descending artery occlusion. *Journal of the American College of Cardiology*.

[14] Bairey C. N., Shah P. K., Lew A. S., Hulse S. (198). Electrocardiographic differentiation of occlusion of the left circumflex versus the right coronary artery as a cause of inferior acute myocardial infarction. *The American Journal of Cardiology*.

[15] Wilson F. N., Johnston F. D., Rosenbaum F. F. (1944). The precordial electrocardiogram. *American Heart Journal*.

[16] Myers G. H., Klein H. A., Stoier B. F. (1944). Correlation of electrocardiographic and pathologic findings in lateral infarction. *American Heart Journal*.

[17] Birnbaum Y., Solodky A., Herz I. (1994). Implications of inferior ST-segment depression in anterior acute myocardial infarction: electrocardiographic and angiographic correlation. *American Heart Journal*.

[18] Engelen D. J., Gorgels A. P., Cheriex E. C. (1999). Value of the electrocardiogram in localizing the occlusion site in the left anterior descending coronary artery in acute anterior myocardial infarction. *Journal of the American College of Cardiology*.

[19] Szymański F. M., Grabowski M., Filipiak K. J (2007). Electrocardiographic features and prognosis in acute diagonal or marginal branch occlusion. *The American Journal of Emergency Medicine*.

[20] Birnbaum Y., Herz I., Sclarovsky S. (1996). Prognostic significance of the admission electrocardiogram in acute myocardial infarction. *Journal of the American College of Cardiology*.

[21] Schamroth L., Schamroth L. (1984). Anterior wall myocardial infarction. *The Electrocardiography of Coronary Artery Disease*.

[22] Dastidar A. (2013). *STEMI Equivalent: are We Missing the STEMIs?*.

[23] Littmann L. (2016). South African flag sign: a teaching tool for easier ECG recognition of high lateral infarct. *The American Journal of Emergency Medicine*.

[24] Lin Y.-Y., Wen Y.-D., Wu G.-L., Xu X.-D. (2019). De Winter syndrome and ST-segment elevation myocardial infarction can evolve into one another: report of two cases. *World Journal of Clinical Cases*.

